# Numerical Modeling of Residual Stresses and Fracture Strengths of Ba_0.5_Sr_0.5_Co_0.8_Fe_0.2_O_3−δ_ in Reactive Air Brazed Joints

**DOI:** 10.3390/ma16237265

**Published:** 2023-11-21

**Authors:** Donat Rudenskiy, Simone Herzog, Lutz Horbach, Nils Christian Gebhardt, Felix Weber, Anke Kaletsch, Christoph Broeckmann

**Affiliations:** Institute for Materials Applications in Mechanical Engineering, RWTH Aachen University, 52062 Aachen, Germany; d.rudenskiy@iwm.rwth-aachen.de (D.R.); l.horbach@iwm.rwth-aachen.de (L.H.); f.weber@iwm.rwth-aachen.de (F.W.); c.broeckmann@iwm.rwth-aachen.de (C.B.)

**Keywords:** BSCF, reactive air brazing (RAB), residual stress, fracture strength, finite element analysis (FEA), representative volume elements (RVEs), multiscale modeling

## Abstract

Reactive Air Brazing (RAB) enables the joining of vacuum-sensitive oxide ceramics, such as Ba_0.5_Sr_0.5_Co_0.8_Fe_0.2_O_3−δ_ (BSCF), to metals in a one-step process. However, damage may form in ceramic or joint during RAB. In this work, experimental microstructure characterization, measurement, and prediction of local material properties using finite element analysis were combined to enlighten these damage mechanisms, which are currently not well understood. Micromechanical simulations were performed using representative volume elements. Cooling simulations indicate that small-sized CuO precipitations are most likely to cause crack initiation in BSCF during cooling. The ball-on-three-balls experiment with porous BSCF samples was analyzed numerically to determine the values of temperature-dependent BSCF fracture stresses. The inversely calibrated fracture stresses in the bulk BSCF phase are underestimated, and true values should be quite high, according to an extreme value analysis of pore diameters.

## 1. Introduction

The joining of ceramic to metal is a difficult task caused by their dissimilar material behavior. Several joining techniques are available for high-temperature applications of ceramic-metal joints, including the field of high-temperature brazing. Here, the so-called “vacuum-active brazing” is mostly used because it can join almost all ceramics and achieves usually very good adhesive strengths [1,2,3]. For the reaction of the active components of the solder (mostly Ti, Hf or Zr), the joining process must take place under cost-intensive vacuum or inert gas atmospheres. The “Reactive Air Brazing” (RAB) process [4], which has been developed since 2004, is a one-step process that can join oxide ceramics with metallic braze alloys in air without pretreatment of the ceramic. RAB alloys rely on a noble metal as the base component. The addition of metal oxides as reactive components lowers the wetting angle of the braze on the ceramic and enables bonding between braze and ceramic [5,6,7]. The most commonly used and investigated braze system is Ag-CuO. It was used to join zirconia to high-temperature steels [8] and join alumina [9] and various perovskite ceramics [10,11,12,13]. In addition to process simplifications, compared to vacuum-active brazing, RAB offers possibilities for joining ceramics that cannot be joined in high vacuum due to this thermodynamic instability. These materials include Ba_0.5_Sr_0.5_Co_0.8_Fe_0.2_O_3−δ_ (BSCF). BSCF and doped variants were used in research as oxygen-permeable membranes for oxygen removal from air [14]. The operating temperatures of such oxygen transport membranes are 800–850 °C. Operation requires a long-term gas-tight and temperature-resistant joint to metallic components. RAB of BSCF is associated with the formation of different reaction layers and can lead to damage of the BSCF ceramic or joint either already during brazing or membrane operation. The damage in BSCF was characterized by a modified microstructure [15], which is responsible for crack initiating in tensile and bending tests [16]. The reaction layers formed on the BSCF and the steel side can be influenced to a certain extent by the process and depend on the Cu concentration in the brazing alloy, the brazing temperature, the temperature-time curve, and the joint pressure [17]. Although the reaction layers cannot be avoided, they can be designed using numerical simulation. A purely experimental approach by quantification and determination of the influence of individual parameters would be extraordinarily time-consuming. Numerical simulation allows for quantitative prediction of the reaction zones’ mechanical properties and their tendency to damage.

In order to study the damage mechanisms during reactive air brazing by micromechanical simulation, temperature-dependent fracture stresses of the BSCF are required. However, these are currently unknown from the literature. The microstructure of porous BSCF and its braze infiltration layers can be captured by finite element representative volume elements (RVEs). An RVE represents a limited geometric region of the microstructure which is considered representative for the microstructure to be analyzed. Investigations of the material’s failure behavior and mechanical properties under consideration of the microstructure including, e.g., pores, grains, or inclusions, were performed using RVE [18]. Considering the cooling after the RAB process, the temperature-dependent properties of materials captured by the RVE are necessary. Furthermore, an exact determination of boundary conditions in terms of macroscopic internal stresses at relevant temperatures is essential. In earlier publications, 2D [12,17] and 3D [19,20] finite element-based macroscopic cooling simulation models of tubular BSCF (or similar ceramic) brazed joints were provided. The micromechanics of matrix-inclusion problems were postulated by Eshelby [21] for geometrically simple RVEs with elliptic inclusions. Since then, RVE modeling has gained popularity in the field of matrix-inclusion composites and porous materials. Here, the generation of RVEs can be achieved using either “real” RVEs derived directly from micrographs or “artificial” RVEs generated by a computer algorithm [22,23,24]. In the literature, RVE techniques were applied to ceramics and ceramic matrix composites to predict elastic material properties [25,26], thermal conductivity [27], thermal shock induced cracks [28], and material strength for porous media [29]. The RVE simulation of internal stresses in WC-Co during cool down after sintering was conducted [30]. It is particularly worth mentioning that in mesoscopic mechanical simulation of slow cooling processes the temperature gradient within the RVE can be neglected. An established multiscale analysis approach uses the results obtained on the RVE length scale to define the effective constitutive material behavior [31]. Another example of multiscale modeling is semi-concurrent simulations which validate the predicted local microstructure properties against the experimentally obtained fracture strength [18]. A bottom-up approach was presented by Pirkelmann et al. [32] using RVEs, which allowed for evaluation of a required ceramic microstructure depending on desired material properties.

In this paper, a multiscale approach is used where both macroscopic numerical models, the ball-on-three-balls (B3B) fracture test model, and the cooling simulation model interact with their respective RVE models at the mesoscopic scale. For this purpose, experimental microstructure characterization, measurement, and prediction of local material properties using finite element analysis (FEA) is used. The macroscopic modeling is performed using 3D FEA. In the cooling simulation, the focus was on modeling the BSCF infiltration zone, as damage in the BSCF is expected to be induced by precipitations located at grain boundary triple points. To investigate the effect of the composition of those precipitation phases in brazed joints, a case study involving two typical compositions is performed. Characterization of the BSCF infiltration zone is carried out using microscopy and nanoindentation. Geometries derived from micrographs are used to create RVE models.

## 2. Materials and Methods

### 2.1. Sample Production and Characterization

Two types of specimens containing BSCF were produced for the current study, the brazed joint samples and the wetting test samples. In addition, three types of mixed oxide reference samples were produced to obtain the corresponding material properties.

#### 2.1.1. Brazed Joint Sample

**Figure 1:**
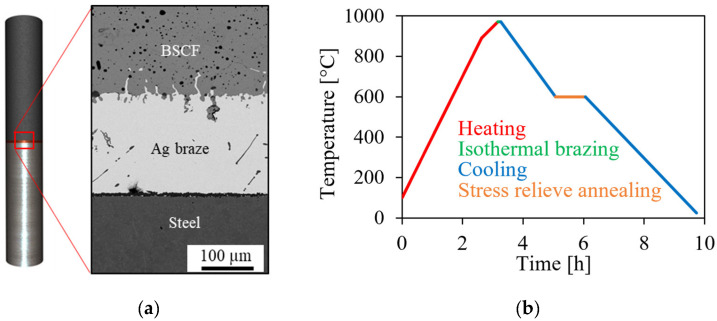
(**a**) Brazed joint specimen (left) and corresponding scanning electron microscope images of the brazed seam (right), (**b**) temperature time profile of brazing.

For production of brazed joint samples as depicted in Figure 1a, BSCF granules supplied by Treibacher Industrie AG (Altofen, Austria) were isostatically pressed into round rods and green and white machined. Final sintering was carried out at 1100 °C for 5 h in air to obtain ceramic components of 20 mm length and 8 mm diameter. Austenitic steel AISI 314 (X15CrNiSi25-21, material no. 1.4841) was machined to the same dimensions. A 70 µm thick silver foil coated with a layer of copper was used as the brazing material, resulting in a total composition of 3 mol% copper. Coins of 8 mm diameter were punched out of the brazing foil. After cleaning all components in isopropanol, two brazed coins were placed between the BSCF and steel bars in a brazing frame and brazed in a muffle furnace (type HT64/16, Nabertherm, Lilienthal, Germany) at 955 °C. During cooling, isothermal annealing at 600 °C for 1 h was introduced to allow creep relaxation of the silver braze and to reduce residual stresses in the joint [12]. The cooling profile is shown in Figure 1b. The sample geometry was designed for flexural testing. In this paper, the brazed joint specimens were used to investigate the thickness of the interfacial layers.

Scanning electron microscope (SEM) images were captured with a JSM 6400 (JEOL GmbH, Freising, Germany) to determine the thickness of braze layer, BSCF infiltration zone, and the mixed oxide layer, see Figure 1a. The braze with a layer thickness of 200 µm consists of pure silver since the copper is oxidized and mainly diffused to the interfaces. The BSCF ceramic forms no sharp interface to the braze. The transition can be described by an infiltration zone in which silver wets and disintegrates. In the infiltration zone, small secondary phases located (triple point phases, TPP) at the triple junctions are found.

#### 2.1.2. Wetting Sample

In order to investigate the properties of the formed TPP phases, a large size of these precipitations is beneficial. Therefore, a wetting test specimen was prepared with a high copper content in the braze and wetting was conducted at a higher temperature than in the brazed joint. Granulated BSCF powder (Treibacher Industrie AG, Althofen, Austria) was uniaxially compacted at 180 MPa to a substrate with 20 mm in diameter and then sintered at 1100 °C for 5 h. Silver and copper powders (Thermo Fisher Scientific, Waltham, MA, USA) were mixed in the appropriate quantities to achieve the desired molar ratio of 14 mol% Cu in silver and were uniaxially pressed at 200 MPa into 6 mm diameter braze pellets. Wetting of the BSCF substrate by the braze pellet and in-situ oxidation of Ag-14Cu to Ag-14CuO was carried out at 1000 °C for 6 min in an air atmosphere, cooling with 300 K/h. A more detailed description can be obtained from [17]. The microstructure of these TPP consists of the four different heterogenous mixed phases BSCF, CoO, Co_3_O_4_, and CuO as investigated in previous work [15].

#### 2.1.3. TPP Reference Samples

Reference samples of the TPP constituent phases CuO, Co_3_O_4_, and CoO were produced to characterize the thermal expansion of each pure phase. The starting powders CuO and Co_3_O_4_ (Thermo Fisher Scientific) were manually mixed with a gel binder (B2-gel, Innobraze, Esslingen am Neckar, Germany) and pressed at 200 MPa into pellets of 20 mm diameter. The CuO and the Co_3_O_4_ pellets were sintered at 760 °C and 850 °C, respectively, each of them for 2 h. To convert the initial Co_3_O_4_ powder to the high-temperature modification CoO [14], a second Co_3_O_4_ pellet was sintered at 1080 °C for 48 h, annealed at 950 °C for 24 h, and finally quenched in water. The temperature-dependent thermal expansion coefficient of CuO, Co_3_O_4_, and CoO was investigated by dilatometry using DIL 402 C (NETZSCH-Gerätebau GmbH, Selb, Germany) in an air atmosphere.

#### 2.1.4. Nanoindentation

**Figure 2:**
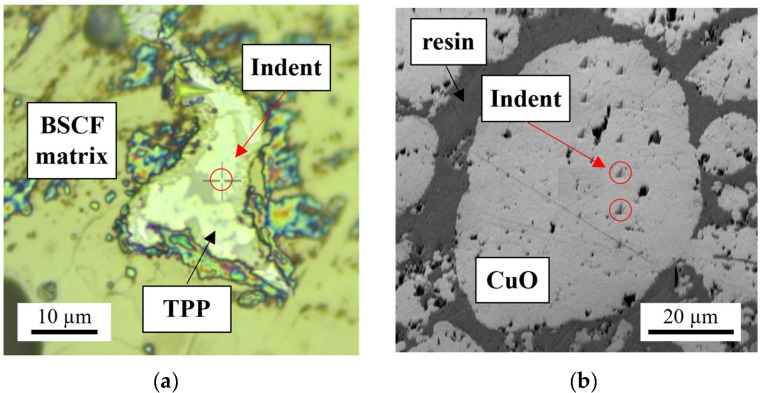
(**a**) Micrograph of the indented TPP and (**b**) SEM image of an embedded CuO particle with indents.

To obtain the elastic constants of bulk BSCF and TPP precipitations, nanoindentation measurements in the cross-sectioned brazed joint specimen were conducted at room temperature using a UNHT^3^ nanoindenter (Anton Paar GmbH, Ostfildern-Scharnhausen, Germany). The specimen was polished with 1 µm diamond suspension. The indents with the maximum force of 1 mN were placed at equal distances of about 3 µm as the whole TTP region was not larger than 10 µm (see Figure 2a). The indentation in the BSCF matrix was performed in a similar manner.

Nanoindentation of the pure CuO reference specimen with a PI Hysitron PI 89 SEM PicoIndenter in Berkovitch geometry (Bruker Corporation, Billerica, MA, USA) was necessary as no experimentally validated data could be found in the literature. The room temperature experiments were performed using continuous measurement techniques. This allows true elastic stiffness to be evaluated in a material that is not fully dense, as was the case with the sintered CuO specimen. Prior to indentation, a small part of the CuO specimen was cut and embedded. After excluding any infiltration of the embedding material in the CuO particles, the sample was ground and polished in order to obtain the cross-sections of spherical CuO particles (see Figure 2b). A series of indents with a maximum force of 10 mN were performed in four randomly selected particles.

### 2.2. Numerical Methods

#### 2.2.1. Multiscale Modeling

**Figure 3:**
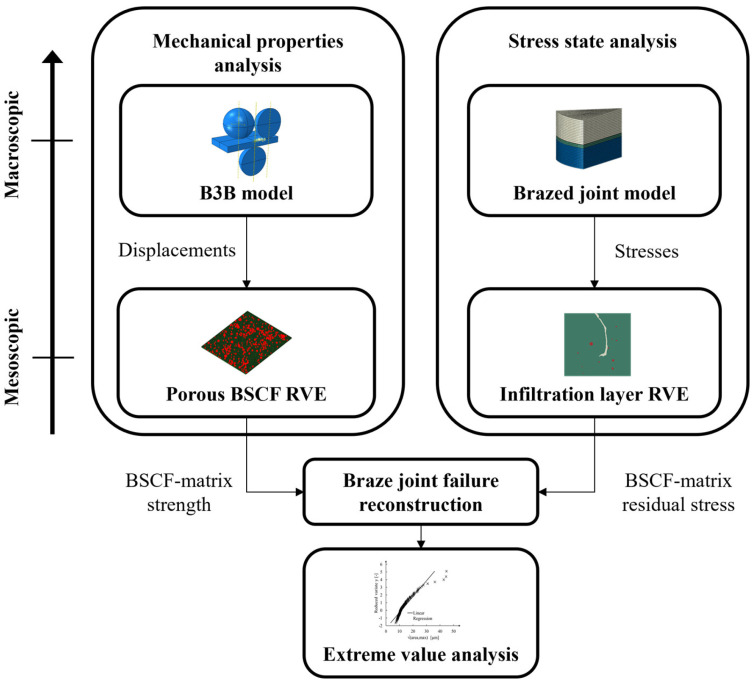
Flowchart showing the hierarchical damage analysis in the BSCF matrix.

In the current study, the top-down approach was used for the hierarchical analysis of brazed joint failure. In this methodology, the modeling process starts by defining the overall behavior of the system and then refines it gradually. The diagram in Figure 3 shows the data flow within the hierarchy levels with corresponding length scales. The modeling is divided into two analyses, both of which use the top-down approach separately. The mechanical properties analysis (Figure 3 left) aims to derive the critical stress values in the BSCF bulk material. At the macroscopic length scale, this part of the analysis includes the B3B virtual experimental model (described in detail in Section 2.2.3) and at the mesoscopic scale the porous BSCF RVE obtained by microstructural characterization of specimens (see Section 3.2.4). Another part of the analysis (Figure 3 right) is aimed at estimating the internal stresses caused by strain incompatibilities in the BSCF infiltration zone during cooling. This part of the analysis uses input in the form of macroscopic residual stress from the brazed joint model (described in detail in Section 2.2.5). The residual stresses in the BSCF matrix that are likely to cause the brazed joint failure are simulated using infiltration layer RVEs. The microstructure of these RVEs was derived from the microstructural analysis of the wetting test specimen. By comparing the BSCF matrix strength with the local stresses, the possible failure scenarios of the brazed joint are assessed. Finally, an extreme value analysis of defect sizes in porous BSCF is performed to validate the numerical results (see Section 2.2.4).

#### 2.2.2. Generation of Representative Volume Elements

**Figure 4:**
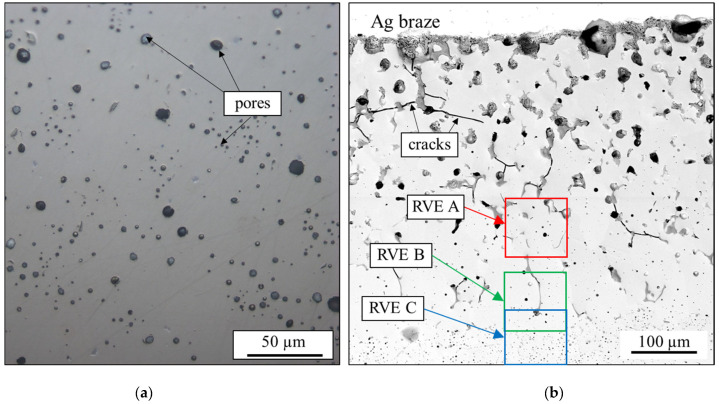
(**a**) Light microscopy image of porous BSCF and (**b**) SEM image of the wetting test sample showing the sections A, B, and C used for creating RVEs.

The micromechanical finite element models were built using micrographs and an in-house Python-based framework [33]. This framework was used for meshing and automated generation of Abaqus FEA input files. Wedge elements of type C3D6 with two Gaussian points from the Abaqus element library were used. The optimal average element size was determined in a convergence study. Two different types of micromechanical models of the respective materials were built: porous BSCF and BSCF containing both pores and TPP inclusions. Figure 4a,b show the light microscopy image of the B3B experimental specimen and the scanning electron microscopy image of a wetting test specimen, respectively. The wetting test sample images were preprocessed using three-color clusters according to the three constituent phases BSCF, TPP, and pores.

#### 2.2.3. Ball-on-Three-Balls Virtual Experiment

**Figure 5:**
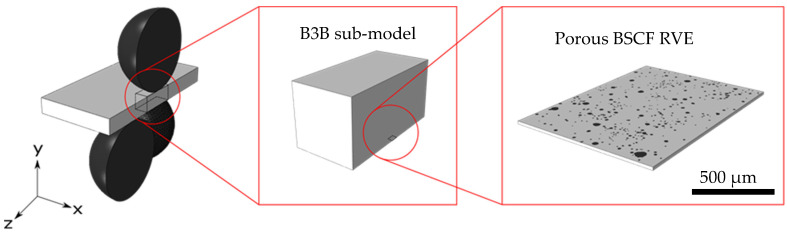
Multiscale model of the virtual B3B experiment.

Inverse modeling was used to evaluate the fracture stresses in the BSCF bulk material at the mesoscopic scale. In order to determine the local mechanical fields, the ball-in-three-balls experiment was modeled by FEA. Here, the boundary conditions were taken from a real B3B experiment [34] carried out by Pfaff et al. [35]. Pfaff et al. determined the BSCF strengths for a range of temperatures between room temperature and 800 °C. BSCF was modeled as purely elastic using temperature-dependent material properties for porous BSCF determined by Huang et al. [36]. The material data used in the B3B macroscopic model can be found in Table S1. The results of the nanoindentation experiment (see Table 1) were used in the mesoscopic porous BSCF model. The hierarchical multiscale modeling of the virtual B3B experiment is depicted schematically in Figure 5. The macroscopic B3B model (Figure 5 left) represents the top layer of the top-down modeling strategy (see Figure 3). In the B3B model, the three balls on the bottom side are fixed in position, and the single ball on the top side is fixed in x- and z-directions and is displacement-controlled in the vertical y-direction. In order to prevent a specimen drift in the x-z plane, the displacement of the specimen center line was limited to the y-direction. To reduce the computational cost, a symmetry boundary condition was applied. This enables the use of a halved geometry of the B3B experiment.

An intermediate modeling layer was required to bridge the length scale difference between the B3B model and the RVE model. This was performed using sub-modeling techniques. In the B3B sub-model (see Figure 5 center), the stresses in the effective volume of the B3B specimen were analyzed. The boundary conditions in the B3B sub-model were defined in terms of surface displacements and taken from the results of the B3B macroscopic model. The dimensions of the B3B sub-model were 10 × 10 × 20 mm^3^, which includes the full thickness of the B3B sample. The evaluation of microstructural stresses was performed in the bottom layer sub-model, which corresponds to the porous BSCF RVE (see Figure 5 right).

The RVE dimensions were 1.02 × 1.35 × 0.02 mm^3^, with the x- and y-dimensions corresponding to the micrograph dimensions (see Figure 4a). The thickness value of 0.02 mm was chosen based on empirical findings from our previous work [33]. The RVE model was positioned on the lower surface of the B3B sub-model representing the maximum tensile stress in the sample. The displacements obtained at the locations of the B3B sub-model, corresponding to the boundaries of the RVE model, were applied to the RVE model as boundary conditions in the x- and y-direction. The loading condition of the RVE model can be described as a biaxial loading and the expected mechanical response is nearly in-plane stress. Using the described multiscale model, virtual experiments were conducted at each temperature corresponding to the real experiment in three steps:

1. The displacement of the single ball on the top side was calibrated using the experimentally determined fracture stresses. Therefore, the displacement in the B3B macroscopic model was varied until the maximum principal stress obtained in the B3B sub-model was equal to the experimental value.
2. The displacements of the calibrated B3B sub-model were mapped to the BSCF RVE as described above.
3. The maximum principal stress value within the BSCF matrix was extracted from the BSCF RVE and considered a strength of the BSCF matrix.

#### 2.2.4. Extreme Value Analysis

The extreme value analysis was necessary because the pores inside the BSCF are underestimated from a statistical point of view. According to the weakest link theory, the maximum defect size is the critical value determining the fracture behavior [37]. Inclusion rating, according to Murakami [38], allows us to estimate the real sizes of crack-initiating defects (here: pores). This enables a correlation of the BSCF matrix strengths obtained by inverse analysis using the virtual B3B experiment (see Section 2.2.3) with the statistical data from the microstructure characterization. As a first step, metallographic sections were taken from the B3B specimens tested by Pfaff et al. [35]. They were ground, polished, and then investigated with an AxioImager M2m Zeiss optical light microscope (Oberkochen, Germany). A total of 162 micrographs of the samples were taken at 100× magnification. For each micrograph, the Feret diameters $d_{Feret}$ were determined by image analysis using ImageJ 1.53f [39] and sorted in ascending order. The parameter $\sqrt{{area}_{max}}=\frac{\sqrt{\pi }\;}{2}\;d_{Feret,max}$ of the respective pores was then derived from their Feret diameters. After evaluating the micrographs, the values of the cumulative distribution function of $\sqrt{{area}_{max}}$ were determined

(1) $$F_{j}=\frac{j}{N+1}$$

By double logarithmization of Equation (1) a linear form of the reduced variates y can be obtained

(2) $$y_{j}=-\ln\left[-\ln\left(F_{j}\right)\right].$$

The linear regression was then carried out to fit a relation of the form

(3) $$\sqrt{{area}_{max}}=a\cdot y+b.$$

For the current extreme value analysis, the effective volume in which the average stress is high enough to cause failure is of interest. The largest pore within this volume in Equation (3) determines the local critical stress in the BSCF matrix. The effective volume of the B3B-test specimens was determined using the virtual experiment model. For calculation, the principle of independent action

(4) $$V_{eff}=\sum_{i=1}^{NE}{\left(\frac{\sigma_{I,i}+\sigma_{II,i}+\sigma_{III,i}}{\sigma_{I,max}}\right)}^{m}\cdot V_{i}$$

was considered. In Equation (4) the effective volume $V_{eff}$ is represented as a sum over all finite elements NE. The volume of each finite element $V_{i}$ is scaled by the stress ratio between the actual principal stresses in element i and the maximal principal stress in the whole model $\sigma_{I,max}$. The power m represents the experimentally derived Weibull modulus. The inspection volume $V_{0}$ represents the total volume considered by micrographs and is estimated by

(5) $$V_{0}=h\cdot S_{0}$$

where $S_{0}$ represents the total inspected area of micrographs. The height h in Equation (5) is estimated as an average maximal Feret diameter:

(6) $$h=\frac{\sqrt{\pi }\;}{2}\frac{\sum d_{Feret,max}}{N}$$

where N=162 is the total number of micrographs inspected. The inspection volume $V_{0}$ is usually smaller than the effective volume $V_{eff}$. For each set of micrographs containing j samples, a corresponding parameter called the return period can be defined by

(7) $$T_{j}=j\cdot \frac{V_{eff}}{V_{0}}.$$

The formulation in Equation (7) is cumulative in nature, similar to Equation (1). The cumulative distribution of maximum pore sizes as a function of data set size can be represented by

(8) $$F_{j}=\frac{T_{j}-1}{T_{j}}.$$

The reduced variates needed for estimation of the real $\sqrt{{area}_{max}}$ parameter in Equation (3) can be calculated by substituting Equation (8) into Equation (2)

(9) $$y_{j}=-ln\left\{-ln\left[\frac{\left(T_{j}-1\right)}{T_{j}}\right]\right\}.$$

Finally, the linear regression coefficients a and b in Equation (3) are obtained by minimizing the least squares errors.

#### 2.2.5. Brazed Joint Modeling

The brazed joint model was built to simulate the internal stresses during the cooling process. According to the top-down multiscale modeling (see Figure 3), RVEs were used to access the stresses in the BSCF infiltration zone at mesoscale, where TPP formation is expected. All TPPs were assumed to be formed at the start of cooling when all components were assumed to be stress- and strain-free. The morphology of the RVE models A, B, and C was obtained using SEM images of the BSCF infiltration zone in the wetting test specimen (see Figure 4b). In the macroscopic model, effective mechanical material properties were used for BSCF and the BSCF infiltration zone, and the mixed oxide layer. The coefficients of thermal expansion used in the macroscopic model can be found in [17]. In RVE models, the elastic BSCF and TPP material properties obtained from nanoindentation experiments were used. The elastic properties of BSCF, Ag braze, and steel used in the macroscopic brazed joint model can be found in Table S1 and the flow properties in Table S2. The respective material properties of the interlayers were approximated using the linear rule of mixtures according to their chemical compositions. For all three RVE models used in the mechanical analysis, two types of TPP material properties were considered in order to investigate the effect of TPP chemical composition on stress. The single-phase TPP model assumes that all TPPs consist of CuO only, while the multi-phase TPP model considers a mixture of CuO, Co_3_O_4_, CoO, and BSCF. In terms of thermal expansion mismatch, the single-phase and multi-phase TPP models represent the worst-case and the best-case scenarios, respectively.

**Figure 6:**
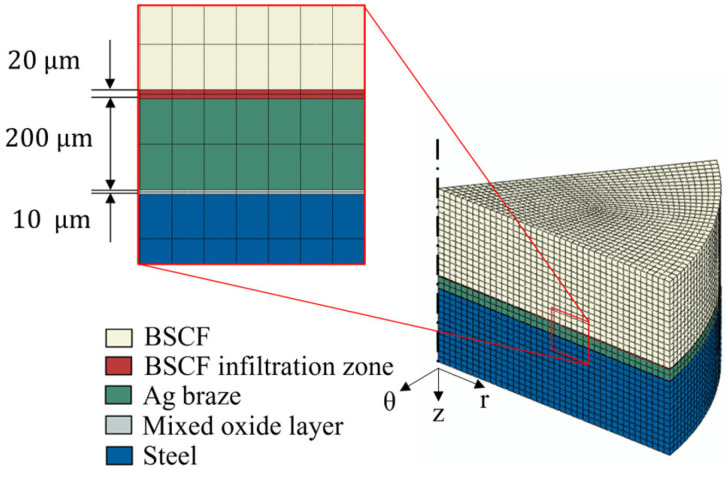
The 3D FE model of the brazed joint with the material assignments.

The geometry model of a macroscopic brazed joint and the phase assignment is shown in Figure 6. The model comprises three coaxial layers representing the BSCF part, Ag braze part, and the steel part, respectively. The BSCF infiltration zone and the mixed oxide layer are modeled at the upper and the lower Ag braze interfaces, respectively. The interfaces share nodes with their neighboring layers, i.e., such effects as delamination were not considered. The BSCF infiltration zone and mixed oxide zone layers were modeled with 2 elements in the vertical direction of the sample for each interface. As high stress gradients within the respective interface layers were expected, fully integrated finite elements with 27 Gauss points were used. The diameters of the cylindrical parts were 8 mm corresponding to the round rod samples (see Section 2.1). The rotational symmetry was used to reduce the size of the numerical model. A 45° sector with a height of 3 mm was modeled, where the height of the BSCF section was 1.5 mm and the remaining 1.5 mm was divided between the BSCF infiltration layer, the Ag braze section, the mixed oxide layer, and the steel section. Symmetric mechanical boundary conditions were applied to both cutting surfaces to ensure the realistic deformation of the 45° sector model. A structured mesh with a total number of 34,816 hexahedral second-order elements (Abaqus code C3D20) was used for all model domains.

In the simulation, the macroscopic brazed joint is cooled from a brazing temperature of 955 °C to room temperature (25 °C) according to the experimental cooling profile (see Figure 1b). Heat transfer from the atmosphere and heat conduction within the sample were neglected. The stress tensors in the BSCF infiltration zone were extracted from the macroscopic model. The principal stresses in axial and radial direction were then introduced into the RVE models as boundary conditions.

## 3. Results and Discussion

### 3.1. Experimental Determination of Model Parameters

#### 3.1.1. Infiltration Zone Microstructural Analysis

**Figure 7:**
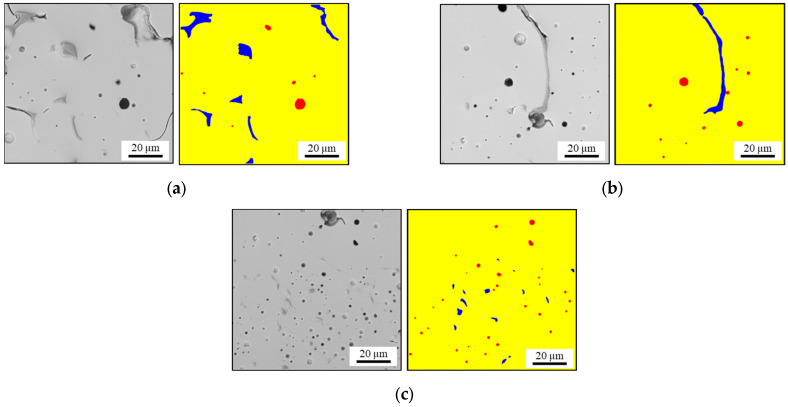
(**a**) SEM images and phase mapping in RVE A, (**b**) in RVE B, and (**c**) RVE C. The color-code is yellow (BSCF), red (pores), and blue (TPP).

In this section, the results of the semi-automated SEM image segmentation and phase assignment are presented. The identified phases shown in Figure 7 were considered in the FEA and were designated as RVE A, RVE B, and RVE C. Morphological features which could not be unambiguously identified or did not fully lie in the cross-sectional plane were not considered in the numerical model. The larger image of the wetting test specimen microstructure is shown in Figure 4b. Detailed inspection shows that both average pore size and TPP size appears to decrease with the distance from the braze seam. This observation was quantitatively confirmed in [40].

#### 3.1.2. BSCF and TPP Material Properties

The elastic material properties of BSCF and TPP were obtained from nanoindentation using the Oliver and Pharr method [41]. The Young’s modulus of BSCF yielded a value of 119.4 ± 3.8 GPa which is almost exactly double the effective value for porous BSCF measured in a macroscopic ring-on-ring test [36]. The measurement values of the TPP were 230.5 ± 50.4 GPa, where the relatively high scatter is evidence that multiple TPP modifications were involved. Based on findings from [15], the TPP modification indented was assumed to be a multi-phase TPP consisting of CuO, Co_3_O_4_, CoO, and BSCF.

**Figure 8:**
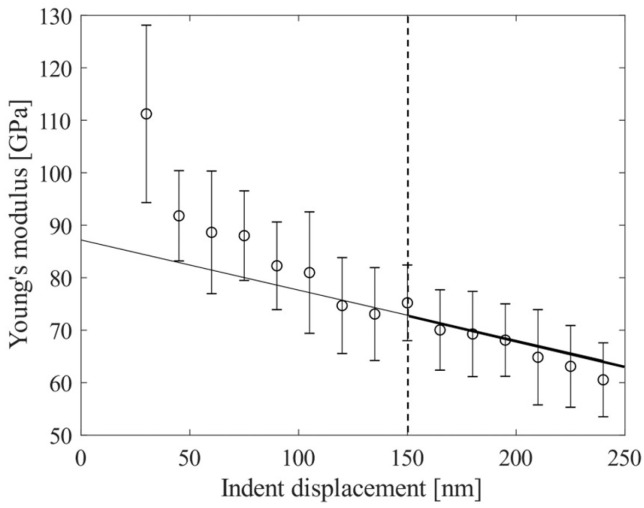
Arithmetic average, standard deviation, and extrapolation of the true CuO Young’s modulus for a porous CuO specimen.

The evaluation of Young’s modulus of CuO, referred to as a single-phase TPP, has required an approach previously used in the indentation of porous bulk or thin films [42]. The graph in Figure 8 shows the results of a continuous Young’s modulus measurement where 19 indents in four different particles were used to extrapolate the true value.

The cutoff indenter displacement was determined at 150 nm. Below this indentation depth the effects of porosity between particles and the interaction with the polymer binder biases the measurements for some particles. The extrapolation value is the y-axis intercept, i.e., the theoretical value for Young’s modulus at zero indent displacement. The obtained 87 ± 8.7 GPa agrees well with the literature data on CuO. Yao et al. obtained 87.907 GPa for CuO at 0 K by using first-principles calculations [43]. Thus, the evaluation method using extrapolation seems to be suitable for the nanoindentation of the porous CuO specimen.

The list of temperature-dependent material properties used in simulation models is provided in Table 1. The high-temperature values of Young’s modulus of CuO were obtained by scaling the room temperature data using the established model for the stiffness reduction in ceramics (10% loss of stiffness per 100 K increase in temperature [44]). For multi-phase TPP and BSCF the scaling of high-temperature stiffness was performed using the experimentally obtained stiffness-temperature relation of porous BSCF [36]. The coefficients of thermal expansion (CTE) of single-phase TPP were obtained by measuring the elongation of CuO sample using dilatometry. The multi-phase TPP coefficients of thermal expansion were calculated using the linear rule of mixtures assuming equal proportions of CuO, Co_3_O_4_, CoO, and BSCF. The Poisson’s ratio of BSCF and Multi-Phase TPP were assumed to equal 0.25. For CuO a value of 0.39 was obtained from literature [43].

### 3.2. Numerical Results

#### 3.2.1. BSCF Bulk Material Strength

The values of temperature-dependent fracture stresses of BSCF as a bulk material (BSCF matrix) represent one of the main goals of the current study. In this section, the results of the inverse analysis using the virtual B3B experiment FE model are presented. The obtained values of the critical maximal principal stress at distinct temperatures are listed in Table 2. All results are approximately an order of magnitude higher than the effective strength of porous BSCF. Qualitatively, the bulk BSCF strengths obtained from the RVE model follow a similar behavior as the B3B results. The BSCF matrix strength at 24 °C, which is below that value for 100 °C, is an exception. However, considering that both values are relatively close to each other, this deviation can be explained by a numerical inaccuracy of the inverse modeling procedure. This error can be usually reduced by considering a larger sample of RVEs. In the current study, a singular RVE was used in order to reduce the numerical effort. Considering another RVE would increase the minimum required simulations by a factor of seven (number of temperatures).

#### 3.2.2. Macroscopic Internal Stresses in Brazed Joint

**Figure 9:**
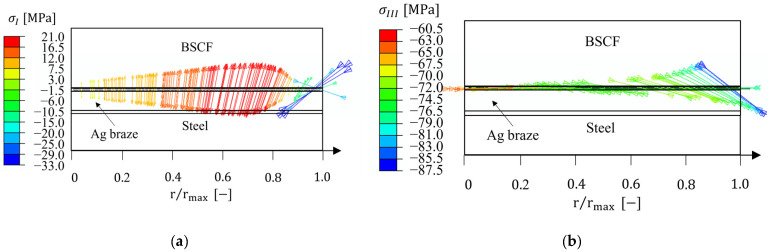
Residual stress eigenvectors in the BSCF infiltration zone at room temperature for (**a**) maximal principal stress and (**b**) minimal principal stress.

This section discusses the macroscopic stresses in the brazed joint due to mismatch of thermal expansion coefficients between the materials in contact. Emphasis is put on the magnitudes of the internal stresses in the BSCF infiltration zone and their spatial directions. The results for BSCF bulk, Ag braze, and steel domains are given in Table S1. The macroscopic stresses presented should be considered as averaged values over the entire thickness of the infiltration zone. Figure 9 shows the maximum and minimum principal stress eigenvectors corresponding to the residual stress state at room temperature. The eigenvectors are helpful for understanding the macroscopic forces acting in the BSCF infiltration zone. It can be seen that macroscopic tensile stresses act in the axial direction and the maximum is at about $r=3/4\;r_{max}$. The stress magnitudes are not sufficient to cause damage in porous BSCF (see critical values in Table 2). The magnitude of compressive residual stress is a factor of four higher than the tensile stresses and the predominant direction is radial (accordingly, the intermediate principal stress is nearly in the circumferential direction). Remarkably, the stress vectors change their direction close to the surface where the compressive stress magnitudes increase. In general, thermally induced compressive stresses at the surface are frequently observed, e.g., after the quenching of metallic alloys during the heat treatment.

**Figure 10:**
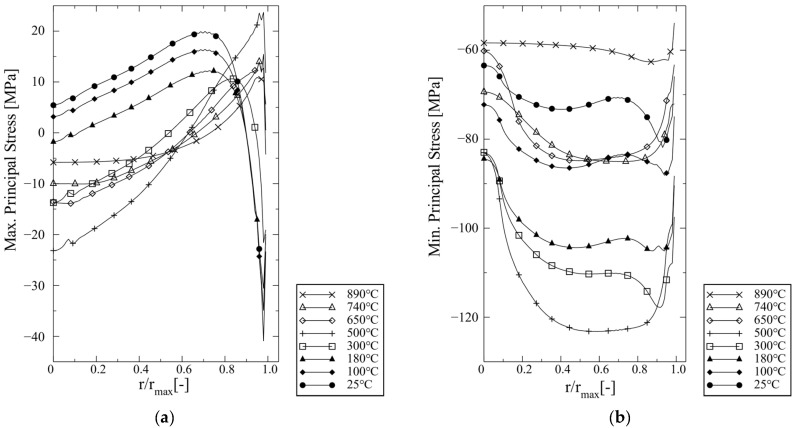
(**a**) Maximum principal and (**b**) minimum stresses plot against the normalized radial distance in the BSCF infiltration zone for distinct temperatures.

The maximum principal ($\sigma_{I}$) and minimum principal stress ($\sigma_{III}$) curves at distinct temperatures during cooling are shown in Figure 10. The $\sigma_{I}$ stress curves behave similarly at temperatures below 500 °C where there is a certain stress peak near $r=3/4\;r_{max}$. At temperatures above 500 °C the tensile stress peaks are located just below the surface (balanced by compressive stresses at the surface). It seems obvious that the change of stress curve is caused by the dwell period at 600 °C. However, in the macroscopic simulation model no creep strains were obtained, but purely time-independent plastic deformation in the Ag braze (see Figure S4 in File S1). The $\sigma_{III}$ curves shown in Figure 10b are compressive during the whole cooling period. Their magnitudes change more than $\sigma_{I}$ during cooling, where the temperature of 500 °C marks a turning point.

#### 3.2.3. Thermally Induced Stresses in BSCF

This section summarizes the results of the numerical study of the internal stresses in the BSCF matrix. Three RVE geometries containing different amounts and distributions of TPP and pores were simulated. The numerical model generation corresponds to the RVE model used in the B3B multiscale simulation for porous BSCF RVE (Section 2.2.3). The radial positions of all RVEs were assumed to be at $r=3/4\;r_{max}$. Furthermore, the RVEs were assumed to be oriented so that the y-direction corresponds to the axial direction of the joint and the x-direction corresponds to the radial direction. In doing so, a biaxial stress state was simulated with $\sigma_{I}$ applied in y-direction and $\sigma_{III}$ in x-direction. This assumption is based on the finding that the directions of principal stress eigenvectors do not change significantly during the cooling at $r=3/4\;r_{max}$. The $\sigma_{II}$ stress acting in z-direction was neglected due to limitations of the 2.5D modeling technique. Effectively, these boundary conditions produce tensile loading in y-direction and compressive loading in x-direction. The corresponding load history was extracted from the macroscopic brazed joint model and the appropriate boundary conditions were applied using interpolation (same temperature profile was used in macro and micro simulations). Therefore, stress values were converted into concentrated forces using the RVE model dimensions.

**Figure 11:**
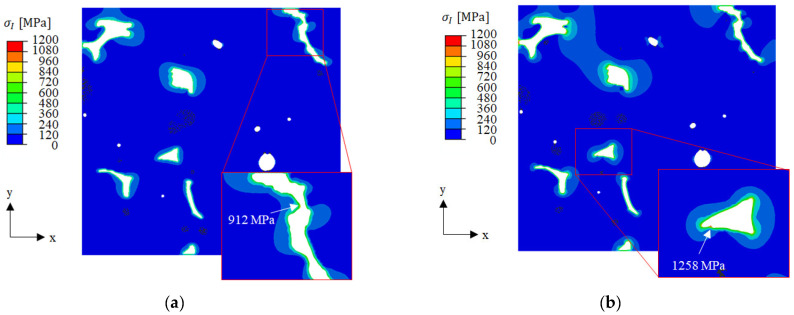

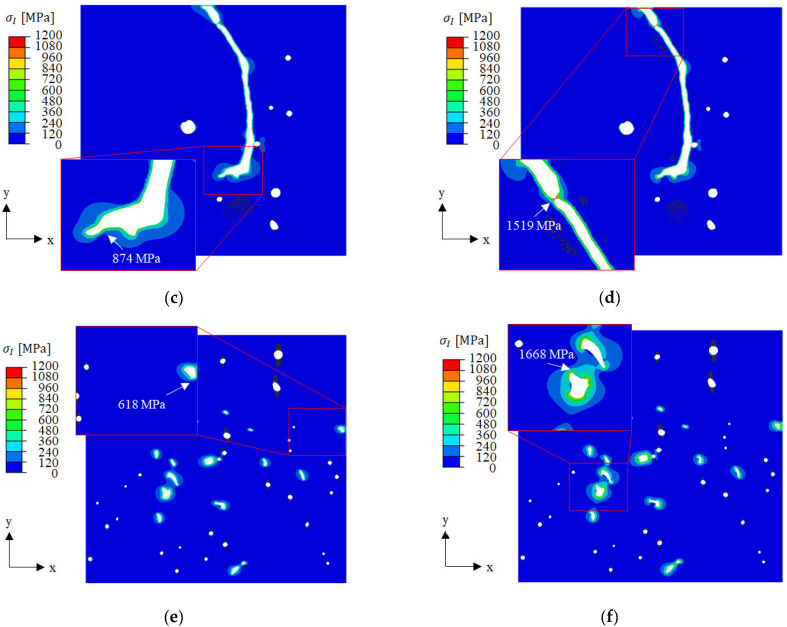
Contour plots of maximum principal stresses in BSCF at room temperature with close-ups showing the locations of peak stresses in (**a**) RVE A multi-phase TPP, (**b**) RVE A single-phase TPP, (**c**) RVE B multi-phase TPP, (**d**) RVE B single-phase TPP, (**e**) RVE C multi-phase TPP, and (**f**) RVE C single-phase TPP. Pores and TPP are shown in white, compare Figure 7.

Figure 11 shows the contour plots of the maximal principal stresses at room temperature. As expected, the highest stresses were found at the end of cooling. In the vicinity of BSCF/TPP phase boundaries, the RVE analysis yielded a steep increase in stress between 100 °C and room temperature (see graphs in Figure S5 in File S1). As expected from the differing material properties, the locally increased BSCF matrix stress is lower in the multi-phase TPP model (left) than in the single-phase TPP model (left). The locations of peak stresses were throughout the BSCF/TPP phase boundaries. Remarkably, the stress peaks of the individual RVE models were found at different locations, see close-ups. This indicates a completely different mechanical material response when the phase composition of the precipitation changes. In multi-phase TPPs, the inclusions of smaller size caused smaller magnitudes of residual stresses which was not the case for single-phase TPP. As expected, the single-phase TPP model (CuO) yielded higher BSCF matrix stresses than the multi-phase model. This is due to a disadvantageous combination of elastic properties and the mismatch of thermal expansion coefficients. However, the critical stress was exceeded in only one out of six simulated cases. The RVE C stress value of 1668 MPa lies ca. 5% above the calculated BSCF material strength of 1599 MPa (see Table 2) and therefore crack initiation can be assumed. In RVE B, the stress peak was about 5% below the critical value, and in RVE A the stress peak was significantly below the critical value.

#### 3.2.4. Extreme Value Analysis

**Figure 12:**
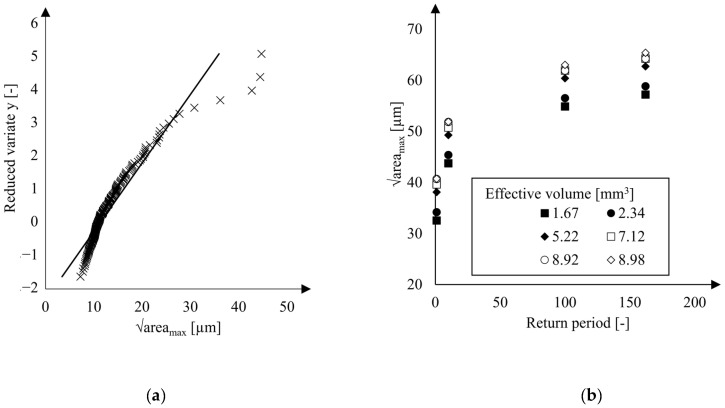
(**a**) Extreme value statistics of pore sizes in BSCF material from B3B-tests and (**b**) predicted $\sqrt{{area}_{max}}$-parameters depending on the calculated effective volumes.

The results of the pore size extreme value statistics were obtained by fitting the model expressed in Equation (3) to measured maximum pore areas. Least squares errors were minimized yielding the plot shown in Figure 12a with regression coefficients a=4.84 and b=11.31.

To account for the temperature dependence of the Weibull modulus in the B3B-tests, the effective volumes $V_{eff}$ were determined, see Table 3. In the calculation according to Equation (4), the macroscopic Young’s moduli of the BSCF were adopted from Huang et al. [36].

The return periods of the effective loaded volumes were calculated using Equation (5). Then, the regression of the extreme value data was used to predict the square root areas corresponding to each of the effective loaded volumes and return periods. The results in Figure 12b show that the critical defect size of the B3B specimens lies in the range of 55–65 µm while the predicted $\sqrt{{area}_{max}}$-parameters increase with increasing effective volumes $V_{eff}$. For each $V_{eff}$, the maximum predicted $\sqrt{{area}_{max}}$-parameter increases with increasing return period T (see Equation (3)). The BSCF strengths were determined using an RVE with a real microstructure derived from an exemplary micrograph. It is inherently difficult to obtain the real $\sqrt{{area}_{max}}$ parameter from one micrograph; this parameter is always below the real value (see Figure 4a). Since smaller defects usually lead to higher strengths, the BSCF strength at the mesoscopic scale is rather underestimated by the inverse modeling procedure. Therefore, the BSCF matrix strengths shown in Table 2 would be accurate if BSCF defects had $\sqrt{{area}_{max}}$-values lower than 30 µm. The additional sources of error can be associated with the $\sqrt{{area}_{max}}$-parameter which might not be sufficient to describe the fracture behavior of BSCF. The local spatial distribution of pores affects the magnitudes of local stress peaks, but it is not considered by the weakest link theory. To overcome this limitation, a 3D geometry of microstructure can be used for finite element models. Possible methods for 3D microstructure reconstruction, especially under consideration of spatial correlations, of the BSCF RVE may be adapted from recent reconstruction methods for heterogeneous materials [45,46,47]. However, it should be considered that serial sectioning of micrographs is very time-consuming and prone to error. For that reason, the artificial RVEs might be preferable.

## 4. Conclusions and Outlook

- Microstructural residual stresses in BSCF-steel joints and temperature-dependent critical stress of bulk BSCF were predicted through the hierarchical multiscale finite element model.
- Mechanical and physical material properties of precipitating triple point phases (TPP) associated with BSCF damage, which were not available in existing literature, were effectively obtained through nanoindentation and dilatometry.
- Mesoscale RVE simulations were used to reconstruct the failure mechanisms. Two material models of TTPs were explored, including multi-phase TPP (CuO, Co_3_O_4_, CoO, and BSCF in equal parts) and single-phase TPP (CuO only).
- Mesoscale cooling simulations revealed that small single-phase TPPs are more likely to cause damage in BSCF compared to larger single-phase TPPs or multi-phase TPPs. Damage in the BSCF occurs during the cooling phase after brazing, typically at temperatures between 100 °C and room temperature. It was observed that crack initiation sites are consistently located at BSCF/TPP phase boundaries.
- To statistically validate the temperature-dependent BSCF fracture stresses, an extreme value analysis based on the weakest link theory was conducted. This analysis revealed that the calibrated fracture strengths obtained from the inverse FEA were underestimated, likely due to limited microstructural information derived directly from a single micrograph.
- Future improvements in the brazing process should focus on promoting the formation of advantageous multi-phase precipitations in the BSCF matrix, thereby enhancing its structural integrity. It is suggested that future work include a 3D microstructure reconstruction to map spatial correlations of precipitation phases and gain better knowledge of the fracture behavior and damage mechanisms of BSCF.

## Figures and Tables

**Table 1:** Material properties of BSCF and TPP.

	BSCF			Multi-Phase TPP		Single-Phase TPP (CuO)	
Temperature [°C]	Young’s Modulus [GPa]	Young’s Modulus [GPa]	CTE [10^−6^ K^−1^]	Young’s Modulus [GPa]	CTE [10^−6^ K^−1^]	Young’s Modulus [GPa]	CTE [10^−6^ K^−1^]
24	119.4	230.5	9.44	230.5	9.44	86.7	4.9
100	99.6	191.7	9.81	191.7	9.81	86.0	5.45
200	88.4	170.3	9.77	170.3	9.77	85.0	6.04
300	84.1	161.9	9.65	161.9	9.65	84.0	6.56
400	96.9	186.6	9.97	186.6	9.97	83.0	6.9
500	99.9	192.5	10.43	192.5	10.43	82.0	718
600	100.7	193.9	11.30	193.9	11.30	81.0	7.61
700	95.4	183.7	12.36	183.7	12.36	80.0	8.39
800	91.8	176.8	13.19	176.8	13.19	79.0	10.19
900	-	-	-	-	-	78.0	-
1000	-	-	-	-	-	77.0	-

**Table 2:** BSCF failure stresses of the BSCF matrix from simulation at distinct temperatures. Porous BSCF strengths from B3B experiment are given for reference.

Temperature [°C]	BSCF Matrix [MPa]	Porous BSCF [MPa] [35]
24	1599	188
100	1620	169
200	732	75
300	708	69
400	674	71
600	895	87
800	1210	108

**Table 3:** Effective volumes in B3B test.

Temperature [°C]	Weibull Modulus [-] [35]	Effective Volume $V_{eff}$ [mm³]
24	8.5	1.67
100	5.4	5.22
200	4.8	7.12
300	4.4	8.92
400	4.8	7.12
600	4.1	8.98
800	7.4	2.34

## Data Availability

The data presented in this study are available in the article and the Supplementary Materials.

## Supplementary Materials

The following supporting information can be downloaded at: https://www.mdpi.com/article/10.3390/ma16237265/s1, Table S1: Elastic properties; Table S2: Flow properties; File S1: Extended evaluation FE modeling.

## Abbreviations

BSCF	Ba_0.5_Sr_0.5_Co_0.8_Fe_0.2_O_3−δ_—Oxygen transport membrane material
TPP	Triple point phase
RAB	Reactive Air Brazing
B3B	ball-on-three-balls (test)
2D/3D	Two dimensional/three dimensional
RVE	representative volume element
FEA	finite element analysis
SEM	Scanning electron microscope

## Mathematical Symbols

$\sqrt{{area}_{max}}$	critical defect size according to Murakami
a, b	linear regression coefficient
$d_{Feret,max}$	maximal Feret diameter in the whole image
$d_{Feret}$	Feret diameter of a pore
$F_{j}$	cumulative distribution of maximum pore sizes
h	estimated height of the inspected micrographs
j	number of micrographs
m	Weibull modulus
N	total number of micrographs inspected
NE	number of finite elements
$r/r_{max}$	relative radial position of in a brazed joint
$S_{0}$	total inspected area of micrographs
$T_{j}$	return period
$\sigma_{I,i}$	maximum principal stress in the volume element i
$\sigma_{I,max}$	maximal principal stress in the whole model
$\sigma_{II,i}$	medium principal stress in the volume element i
$\sigma_{III,i}$	minimal principal stress in the volume element i
$V_{0}$	inspection volume
$V_{eff}$	effective volume
$V_{i}$	volume of each finite element
y	reduced variate

## References

[1] Singh M., Ohji T., Asthana R., Mathur S., Singh M. (2011). Ceramic Integration and Joining Technologies: From Macro to Nanoscale.

[2] Srivastava A.K., Sharma A. (2017). Advances in Joining and Welding Technologies for Automotive and Electronic Applications. Materials.

[3] Moret F., Eustathopolous N. (1993). Ceramic to metal direct brazing. J. Phys. IV Fr..

[4] Weil K.S., Kim J.Y., Hardy J.S. (2005). Reactive Air Brazing: A Novel Method of Sealing SOFCs and Other Solid-State Electrochemical Devices. Electrochem. Solid-State Lett..

[5] Darsell J.T., Weil K.S. (2007). The effect of palladium additions on the solidus/liquidus temperatures and wetting properties of Ag-CuO based air brazes. J. Alloys Compd..

[6] Friant J.R., Meier A., Darsell J.T., Weil K.S. (2012). Transitions in Wetting Behavior Between Liquid Ag-CuO Alloys and Al_2_O_3_ Substrates. J. Am. Ceram. Soc..

[7] Kim J.Y., Hardy J.S., Scott Weil K. (2005). Effects of CuO Content on the Wetting Behavior and Mechanical Properties of a Ag-CuO Braze for Ceramic Joining. J. Am. Ceram. Soc..

[8] Tillmann W., Anar N.B., Wojarski L. (2020). Mechanical behavior of reactive air brazed (RAB) Crofer 22 APU-Al_2_O_3_ joints at ambient temperature. SN Appl. Sci..

[9] Cao J., Si X., Li W., Song X., Feng J. (2017). Reactive air brazing of YSZ-electrolyte and Al_2_O_3_-substrate for gas sensor sealing: Interfacial microstructure and mechanical properties. Int. J. Hydrogen Energy.

[10] Zhang Y., Liu T., Zhang J., Wu C., Lu X., Ding W. (2017). Induction brazing BaCo_0.7_ Fe_0.2_Nb_0.1_O_3−δ_ membrane tubes to steel supports with Ag-based filler in air. J. Membr. Sci..

[11] Hardy J.S., Kim J.Y., Weil K.S. (2004). Joining Mixed Conducting Oxides Using an Air-Fired Electrically Conductive Braze. J. Electrochem. Soc..

[12] Bobzin K., Öte M., Wiesner S., Kaletsch A., Broeckmann C. (2014). Characterization of Reactive Air Brazed Ceramic/Metal Joints with Unadapted Thermal Expansion Behavior. Adv. Eng. Mater..

[13] Erskine K.M., Meier A.M., Pilgrim S.M. (2002). Brazing perovskite ceramics with silver/copper oxide braze alloys. J. Mater. Sci..

[14] Han N., Shen Z., Zhao X., Chen R., Thakur V.K. (2022). Perovskite oxides for oxygen transport: Chemistry and material horizons. Sci. Total Environ..

[15] Ehle L.C., Richter S., Herzog S., Broeckmann C., Mayer J. (2020). Identification of Cu-Co-oxide phases of reactive air brazed Ba_0.5_Sr_0.5_Co_0.8_Fe_0.2_O_3−δ_-Ag-14CuO joints by EBSD, EPMA and TEM diffraction. IOP Conf. Ser. Mater. Sci. Eng..

[16] Kaletsch A. (2016). Reaktivlöten von Perowskit-Stahl-Verbunden und deren Alterungsbeständigkeit in oxidierender Atmosphäre.

[17] Kaletsch A., Bezold A., Pfaff E.M., Broeckmann C. (2012). Effects of Copper Oxide Content in AgCuO Braze Alloy on Microstructure and Mechanical Properties of Reactive-Air-Brazed Ba_0.5_Sr_0.5_Co_0.8_Fe_0.2_O_3−δ_ (BSCF). J. Ceram. Sci. Technol..

[18] Budarapu P.R., Zhuang X., Rabczuk T., Bordas S.P. (2019). Multiscale modeling of material failure: Theory and computational methods. Advances in Crystals and Elastic Metamaterials, Part 2.

[19] Kiebach R., Engelbrecht K., Kwok K., Molin S., Søgaard M., Niehoff P., Schulze-Küppers F., Kriegel R., Kluge J., Hendriksen P.V. (2016). Joining of ceramic Ba_0.5_Sr_0.5_Co_0.8_Fe_0.2_O_3_ membranes for oxygen production to high temperature alloys. J. Membr. Sci..

[20] Zhang J., Zhang J., Li L., Zhang C., Zhang Y., Lu X. (2019). Stress analysis of the brazing joints of tubular ceramic oxygen-permeable membranes and metal supports. Ceram. Int..

[21] Eshelby J.D., Markenscoff X., Gupta A. (2006). The determination of the elastic field of an ellipsoidal inclusion, and related problems. Collected Works of J. D. Eshelby.

[22] Liu Y., Steven Greene M., Chen W., Dikin D.A., Liu W.K. (2013). Computational microstructure characterization and reconstruction for stochastic multiscale material design. Comput. Aided Des..

[23] Bostanabad R., Zhang Y., Li X., Kearney T., Brinson L.C., Apley D.W., Liu W.K., Chen W. (2018). Computational microstructure characterization and reconstruction: Review of the state-of-the-art techniques. Prog. Mater. Sci..

[24] Swaminathan S., Ghosh S., Pagano N.J. (2006). Statistically Equivalent Representative Volume Elements for Unidirectional Composite Microstructures: Part I—Without Damage. J. Compos. Mater..

[25] Chen Q., Zhao F., Jia J., Zhu C., Bai S., Ye Y. (2022). Multiscale simulation of elastic response and residual stress for ceramic particle reinforced composites. Ceram. Int..

[26] Horny D., Schukraft J., Weidenmann K.A., Schulz K. (2020). Numerical and Experimental Characterization of Elastic Properties of a Novel, Highly Homogeneous Interpenetrating Metal Ceramic Composite. Adv. Eng. Mater..

[27] Carneiro P.M., Maceiras A., Nunes-Pereira J., Silva P.D., Silva A.P., Baudín C. (2021). Property characterization and numerical modelling of the thermal conductivity of CaZrO_3_-MgO ceramic composites. J. Eur. Ceram. Soc..

[28] Gong Z., Guan K., Rao P., Zeng Q., Liu J., Feng Z. (2021). Numerical Study of Thermal Shock Damage Mechanism of Polycrystalline Ceramics. Front. Mater..

[29] Zimina V.A., Smolin I.Y. (2022). The Modeling of the Fracture of Three-Phase Ceramic Composite. Procedia Struct. Integr..

[30] Kayser W., van Kempen S., Bezold A., Boin M., Wimpory R., Broeckmann C. (2019). Numerical investigation of the WC re-precipitation impact on the residual stress state in WC20 wt.-%Co hardmetal. Int. J. Refract. Met. Hard Mater..

[31] Raju K., Tay T.-E., Tan V.B.C. (2021). A review of the FE2 method for composites. Multiscale Multidiscip. Model. Exp. Des..

[32] Pirkelmann S., Raether F., Seifert G. (2022). Top-down material design of multi-phase ceramics. Open Ceram..

[33] Gebhardt C., Trimborn T., Weber F., Bezold A., Broeckmann C., Herty M. (2020). Simplified ResNet approach for data driven prediction of microstructure-fatigue relationship. Mech. Mater..

[34] Börger A., Supancic P., Danzer R. (2002). The ball on three balls test for strength testing of brittle discs: Stress distribution in the disc. J. Eur. Ceram. Soc..

[35] Pfaff E.M., Oezel M., Eser A., Bezold A., Pyzik A.J., Boccaccini A.R., Dogan F., Lin H.-T., Belharouak I., Marra J.C., Tritt T.M., Sekino T., Katoh Y. (2014). Reliability of Ceramic Membranes of BSCF for Oxygen Separation in a Pilot Membrane Reactor. Ceramics for Environmental and Energy Applications II: A Collection of Papers Presented at the 10th Pacific Rim Conference on Ceramic and Glass Technology, Coronado, CA, USA, 2–6 June 2013.

[36] Huang B.X., Malzbender J., Steinbrech R.W., Singheiser L. (2010). Discussion of the complex thermo-mechanical behavior of Ba_0.5_Sr_0.5_Co_0.8_Fe_0.2_O_3−δ_. J. Membr. Sci..

[37] Munz D., Fett T. (1989). Mechanisches Verhalten Keramischer Werkstoffe.

[38] Murakami Y. (2002). Metal Fatigue: Effects of Small Defects and Nonmetallic Inclusions.

[39] Schneider C.A., Rasband W.S., Eliceiri K.W. (2012). NIH Image to ImageJ: 25 years of image analysis. Nat. Methods.

[40] Herzog S., Boussinot G., Kaletsch A., Apel M., Broeckmann C. (2022). Microstructure coarsening in Ba_0.5_Sr_0.5_Co_0.8_Fe_0.2_O_3−δ_ during reactive air brazing. J. Eur. Ceram. Soc..

[41] Oliver W.C., Pharr G.M. (1992). An improved technique for determining hardness and elastic modulus using load and displacement sensing indentation experiments. J. Mater. Res..

[42] Chen Z., Wang X., Bhakhri V., Giuliani F., Atkinson A. (2013). Nanoindentation of porous bulk and thin films of La_0.6_Sr_0.4_Co_0.2_Fe_0.8_O_3−δ_. Acta Mater..

[43] Yao B., Zhou X., Liu M., Yu J., Cao J., Wang L. (2018). First-principles calculations on phase transformation and elastic properties of CuO under pressure. J. Comput. Electron..

[44] Wachtman J.B., Cannon W.R., Matthewson M.J. (2009). Mechanical Properties of Ceramics.

[45] Bargmann S., Klusemann B., Markmann J., Schnabel J.E., Schneider K., Soyarslan C., Wilmers J. (2018). Generation of 3D representative volume elements for heterogeneous materials: A review. Prog. Mater. Sci..

[46] Seibert P., Ambati M., Raßloff A., Kästner M. (2021). Reconstructing random heterogeneous media through differentiable optimization. Comput. Mater. Sci..

[47] Robertson A.E., Kalidindi S.R. (2022). Efficient generation of anisotropic N-field microstructures from 2-point statistics using multi-output Gaussian random fields. Acta Mater..

