# Supplementary material for: Numerical Modeling of Residual Stresses and Fracture Strengths of Ba0.5Sr0.5Co0.8Fe0.2O3−δ in Reactive Air Brazed Joints
Source: Materials (Basel). 2023 Nov 21;16(23):7265. doi: 10.3390/ma16237265 (PMC10707528; doi:10.3390/ma16237265)
Supplement: Supplementary file 1 [file materials-16-07265-s001.zip › materials-2665511-supplementary-File 1.pdf]

File S1. Extended evaluation of FE models

Macroscopic shrinkage in braze joints

The shrinkage behavior of braze joint was analyzed using the relative total displacement field. The material expansion at brazing temperature was taken as a reference. Figure S1 shows the contour plot of total displacements at room temperature representing the final stage. Surprisingly the simulation results show ca. 13% higher shrinkage in BSCF than in steel. The thermal expansion coefficient of BSCF used in simulation is lower over the whole simulated temperature range. The difference in the range from 2% smaller at 800°C to 22% smaller at 400°C. The detailed stress results are presented in the following successive order: fist the stresses in BSCF and steel then the stresses and the deformations in the Ag braze.

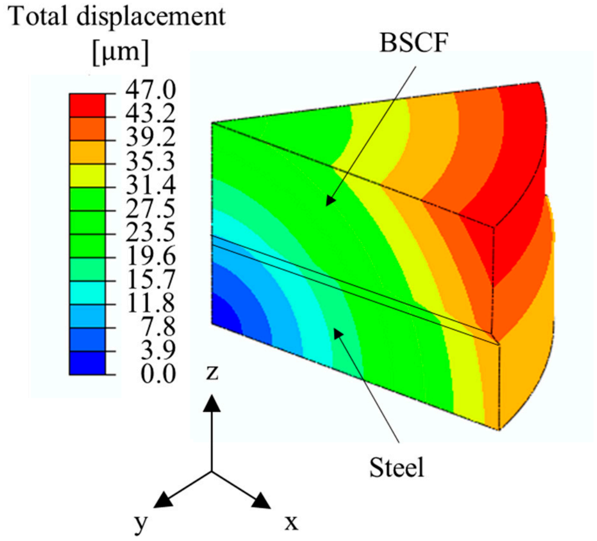

Figure S1 Total displacements in the brazed joint after cooling at room temperature. A scaling factor of 20 is used for better illustration.

Stresses in BSCF and steel

In order to assess the stress state in bulk materials the hydrostatic components of the stress tensor are considered. **Figure S2** shows the contour plots of hydrostatic stresses in the BSCF and steel model sections. The coordinate axes are normalized from zero to one so that one in the radial direction corresponds to the outer edge of either BSCF or steel cylinder and zero corresponds to the center of those cylinders. In the height direction the value of one corresponds to the full height of the braze joint 3D model (1.5 mm). The stresses were evaluated for distinct remarkable temperatures from 890°C which is 60 K below the brazing temperature (955°C) to room temperature. Between the evaluation step at 650°C and at 500°C is the holding ramp which is at 600°C, so that the results for 650° correspond to the state before holding and the results for 500°C after the holding. The maximum tensile hydrostatic stress of ca. 100 MPa was obtained in steel at the outer edge very close to Ag braze the at the temperature of 740°C. The maximal compressive stress of ca. -150 MPa was obtained nearly at the same position and also at the same temperature. The results show compressive stresses in BSCF until 180°C then and stress state changes to tensile. The stress results in steel show a directly opposite behavior; the tensile stresses in the steel core switch to compressive at around 100°C whereas the outward still retain moderate compressive stress. More detailed residual stress analysis is done in BSCF using maximum principal stresses. The contour plot showing the residual stress distribution at

room temperature in the BSCF material section can be found in **Figure S3**. The BSCF infiltration layer which is below the BSCF section is not included in the contour plot. Tensile residual stresses are obtained in the most parts of the simulated BSCF segment. At the cylindrical surface of BSCF the stresses magnitudes are minimal (below 1 MPa). The tensile residual stress magnitudes are very moderate. At the vertical position 0.5 mm away from the BSCF infiltration layer no residual stresses are present. The peak value is about 24 MPa and is located at the radial position around ca. $3/4\ r_{max}$ directly above the BSCF infiltration layer.

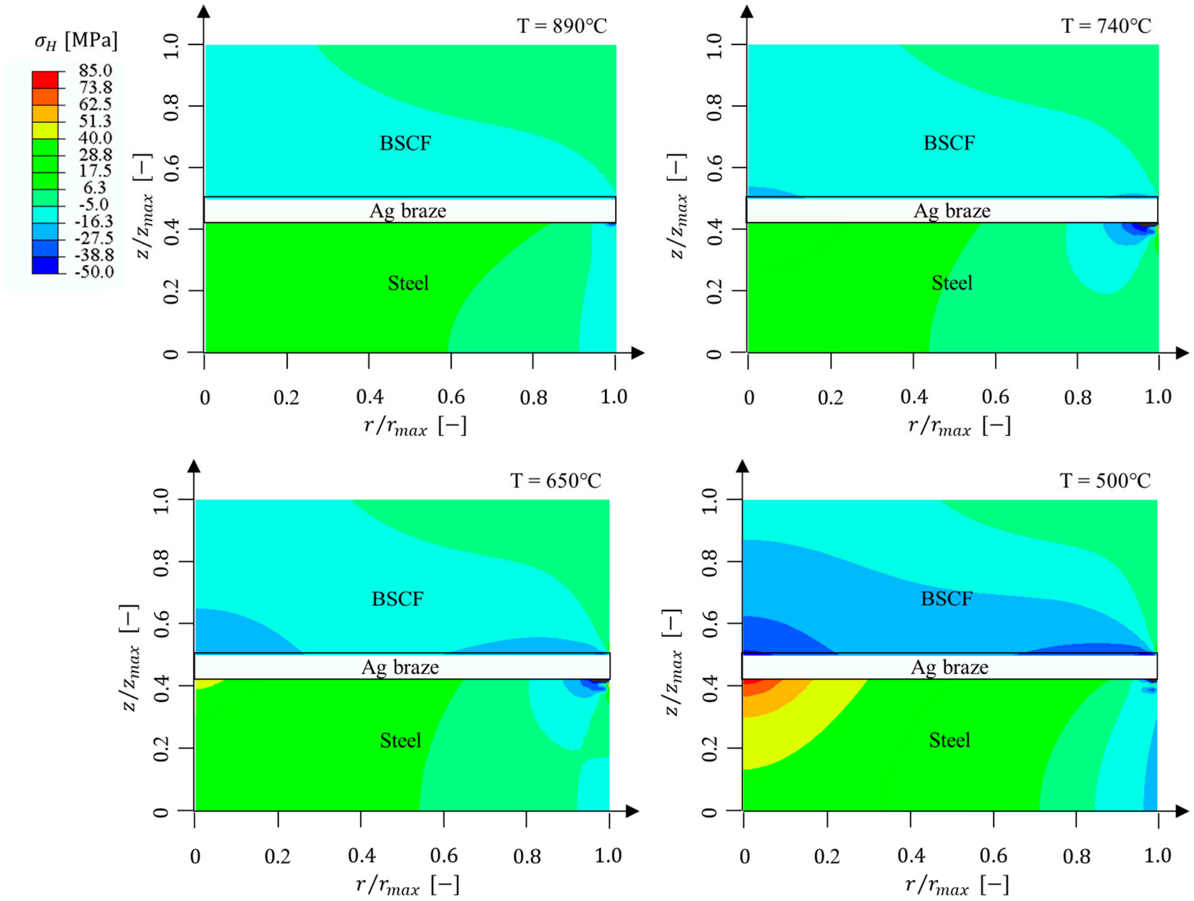

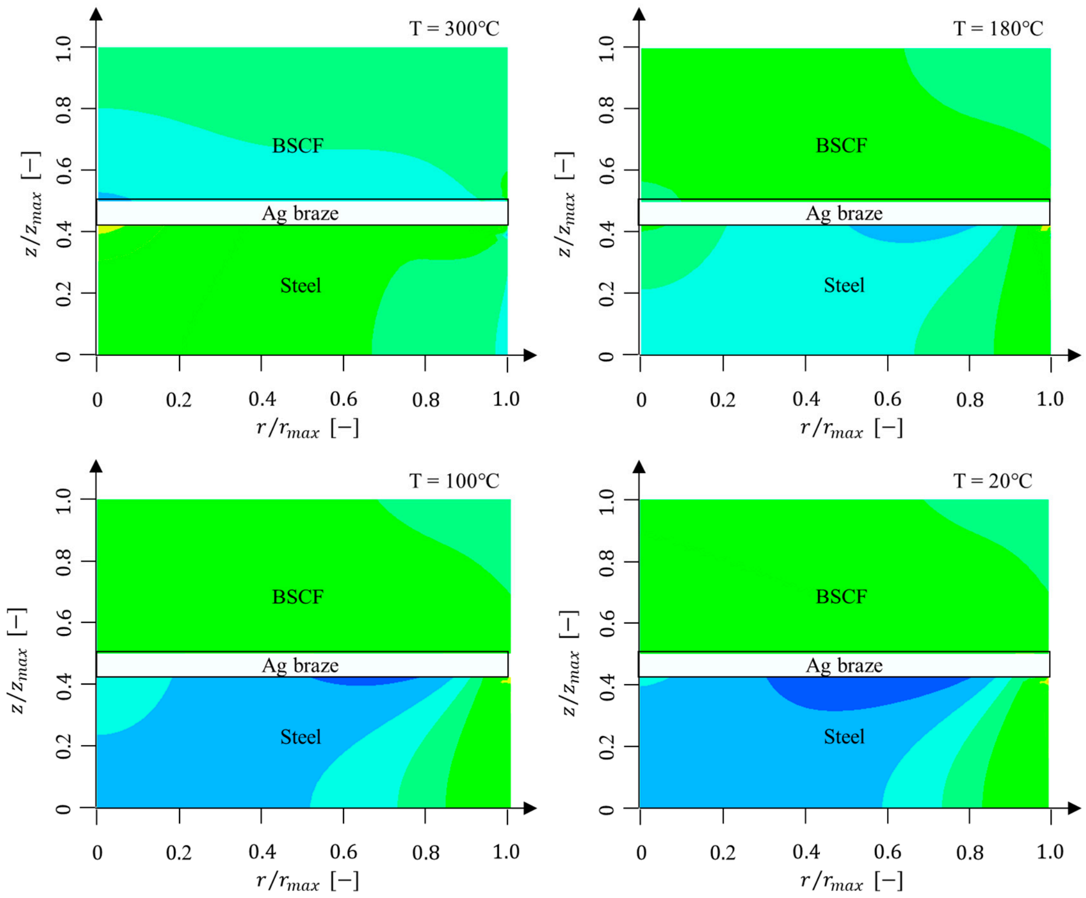

Figure S2 Hydrostatic pressure in BSCF and steel at distinct temperatures during cooling.

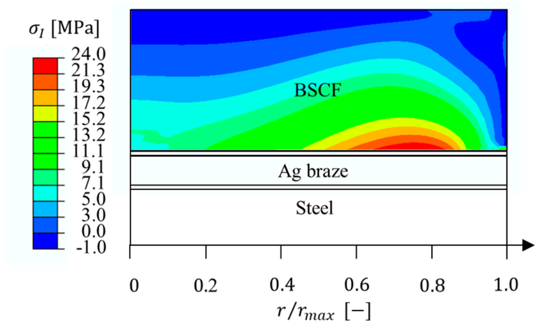

Figure S3 Contour plot of maximum principal stresses in BSCF at room temperature.

Plastic deformation in Ag braze

Ag braze is an easily deformable material, therefore, plastic strain magnitudes during the cooling were evaluated (***Figure S4***). The maximal principal strains which does not depend on the coordinate system choice were considered. In the simulation the plastic deformation onset is just directly at the beginning of the cooling. However, significant plastic strains start to develop around 850°C. Below 500°C almost no additional plastic flow occurs during cooling. Two overlaid strain fields can be identified in the contour plots of ***Figure S4***. The strain at the mixed oxide layer interface is concentrated in the outer edge where the magnitude of ca. 3 % stain is considerably high. At the BSCF infiltration zone side the plastic strain with the magnitude around 2 % is constant from $1/2\ r_{max}$ to $8/9\ r_{max}$. Although creep behavior of Ag braze was modelled, the simulation did not yield creep at any temperature.

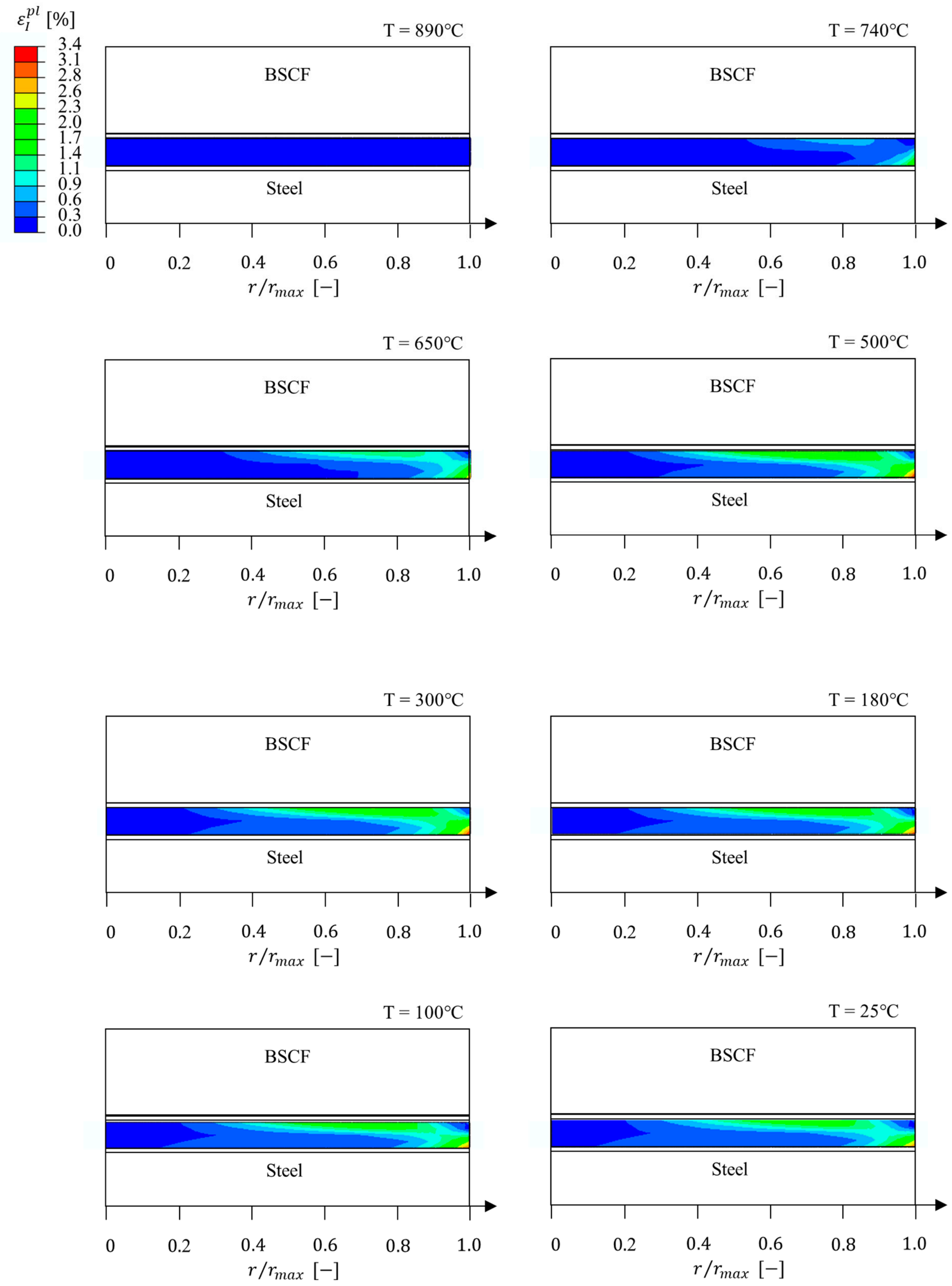

Figure S4 Maximum principal plastic strain in the Ag braze for distinct temperatures during the cooling.

Stress over time in BSCF matrix

Figure S5 shows the curves of local maximum principal stresses in BSCF phase during the cooling process for single-phase and multi-phase TPP model. The positions at which the values were evaluated correspond to previously identified maximally stresses locations. For both TPP models the curves are qualitatively similar. A high increase in the first cooling phase until the holding temperature (600°C) and less increase after the holding temperature. Interestingly, the maximum stresses tend to decrease between 400°C and 300°C which is in a good agreement with the TPP thermal expansion temperature dependency characteristics. Between 250°C and room temperature the stress slope gets steeper reaching its maximum at room temperature. The maximum stress values over time for RVE A and RVE B differ little but the values for RVE C are shifted down by ca. 50-60 MPa.

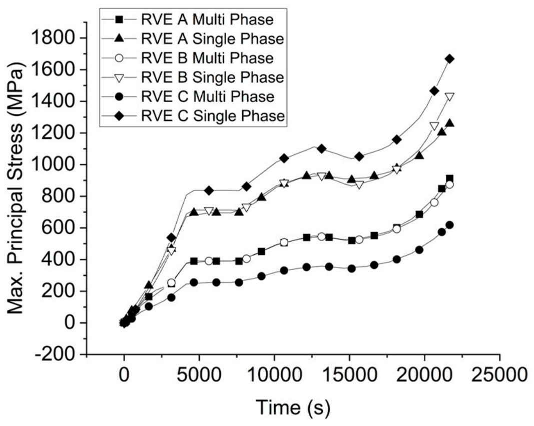

Figure S5 The plot of local maximum principal stresses over time for single-phase TPP model and multi-phase TPP model.
